# Commentary: The treatment of chemotherapy-induced peripheral neuropathy: a review of current management options and a potential role for scrambler therapy

**DOI:** 10.3389/fpain.2025.1677861

**Published:** 2025-09-02

**Authors:** Giuseppe Marineo

**Affiliations:** Delta Delta Research & Development, University of Rome Tor Vergata, Rome, Italy

**Keywords:** scrambler therapy, CIPN, chronic pain, cancer pain, neuropathic pain

A Commentary on The treatment of chemotherapy-induced peripheral neuropathy: a review of current management options and a potential role for scrambler therapy By Aboumerhi H, Vucetic H, Gruenzel A, Moftakhar B, Gupta M, Rao SK and Staudt MD (2025). Front. Pain Res. 6:1607102. doi: 10.3389/fpain.2025.1607102

## 1 Introduction

Scrambler Therapy® is a therapy developed specifically for chronic neuropathic and oncological pain that does not respond to other treatments. Numerous non-profit clinical studies by independent researchers have brought this therapy to the attention of the international scientific and medical community. However, the very nature of nonprofit studies imposes limitations due to costs not covered by an external sponsor. Furthermore, especially in the early stages of the therapy's dissemination, knowledge of this treatment and the possibility of providing adequate training to clinical researchers was very limited. The result was that some publications were produced with unintentional biases introduced by investigation methodologies and treatment procedures that were in stark contrast to the correct use of the therapy (1–3) and standard protocols registered with the FDA.

The purpose of this General Commentary is to highlight the critical issues in the article under review and provide adequate supporting information to restore a correct analysis of the data.

### 1.1 Incompatibility of double-blind studies and methodological alternatives

There is still the need for RCTs, but the proper use of ST, that being operator-dependent, allows only for a partial double-blind or single-blind trial design. Attempts to do a complete double-blind automatically determine substantial changes in the standard treatment user protocol. These changes prevent the operator from following the normal procedures (https://www.scramblertherapy.org/10_basic_-rules.htm) registered in the healthcare authorizations that needed to guarantee the efficacy and safety of the treatment. So, this choice can fully erase or certainly extremely reduce the efficacy of the treatment, consequently invalidating the scientific data. Double-blind studies should therefore never be included in reviews, or alternatively it should be clarified that the data from such studies are not to be considered reliable.

### 1.2 Electrode placement

The authors write: “Electrodes are placed on the skin at the pain site and along corresponding dermatomes.” In reality, the electrodes should be applied as per the instructions registered with the FDA, outside the area of pain, at the edge of the pain, even outside the dermatome primarily affected. Otherwise, the pain may temporarily increase, or the treatment may be ineffective.

### 1.3 Scrambler therapy: mechanisms and indications

In the section “Scrambler therapy: mechanisms and indications,” two theories are mistakenly considered separate: that of information “overwriting pain” and the effects on neuroplasticity. In reality, the functioning model of Scrambler Therapy is based on neuroplasticity induced by the synthetic information of “no pain” (4).

### 1.4 Some patients may experience a recurrence of pain after a period

The stability of treatment results over time depends on certain fundamental conditions:

(1) Pain that will manifest after the treatment due to the progression of neurological damage cannot be treated in advance. It is therefore normal to require follow-up treatments if the progression of neurological damage requires it.
(2) In acute pain that may overlap with the chronic component (typically incident pain), Scrambler Therapy acts as a symptomatic therapy, since acute pain is physiological and not pathological. For this reason, Scrambler Therapy in the treatment of acute pain does not involve a treatment cycle but rather use as needed.
(3) The key to this treatment, and to the duration of the results over time, is the systematic elimination of pain in each application. If this criterion is not met, the results will be lower than the actual potential of the therapy (5).

### 1.5 Safety

The article suggests the possibility of using Scrambler Therapy in patients with implanted stimulators. It should be noted that this is a clear contraindication (even if the implanted devices are turned off) listed in the instruction manuals and registered with the FDA.

## 2 Standardization

The only standardization that should be considered is that of the protocols and treatment methods described in the instruction manuals registered with the FDA and in official training courses. In this case, the variability of results in clinical studies and hospital practice would be considerably reduced.

## 3 Discussion

The article “The treatment of chemotherapy-induced peripheral neuropathy: a review of current management options and a potential role for scrambler therapy” inherited previous issues of incorrect information regarding methods, protocols, and standards of use. There are therefore biases in the analysis carried out by the authors, which have required corrective information.

The application of this information in the future will avoid the risk of further unintentionally incorrect analyses.
